# Understanding stigma of dementia during COVID-19: a scoping review

**DOI:** 10.3389/fpsyt.2024.1261113

**Published:** 2024-03-27

**Authors:** Juanita-Dawne R. Bacsu, Raymond J. Spiteri, Kate Nanson, Zahra Rahemi, Claire Webster, Myrna Norman, Chantelle Stone

**Affiliations:** ^1^ School of Nursing, Thompson Rivers University, Kamloops, BC, Canada; ^2^ Department of Computer Science, University of Saskatchewan, Saskatoon, SK, Canada; ^3^ School of Nursing, Clemson University, Clemson, SC, United States; ^4^ Caregiver Crosswalk Inc, Montreal, QC, Canada; ^5^ Engagement of People with Lived Experience of Dementia (EPLED), Maple Ridge, BC, Canada; ^6^ Department of Psychology, Thompson Rivers University, Kamloops, BC, Canada

**Keywords:** COVID-19, dementia, stigma, stereotypes, prejudice, negative attitudes, discrimination

## Abstract

**Introduction:**

Stigma of dementia is one of the greatest challenges for people living with dementia. However, there is little research on the different types of stigma of dementia in the COVID-19 pandemic. The purpose of this scoping review is to synthesize the existing literature on dementia-related stigma (self, public, and structural stigma), during the pandemic.

**Methods:**

Guided by Arksey and O’Malley’s scoping review framework and PRISMA guidelines, CINAHL, EMBASE, Google Scholar, Medline, PsycINFO, and Web of Science were searched for English language literature from January 2020 to June 2023. Inclusion criteria consisted of peer-reviewed, original research articles addressing stigma of dementia during the COVID-19 pandemic. Thematic analysis was used to analyze the data and steps were taken to ensure rigor.

**Results:**

Fifteen articles met our inclusion criteria. Four primary themes were identified including: 1) COVID-19 stereotypes and assumptions of dementia; 2) human rights issues and deprived dignity; 3) disparate access to health services and supports; and 4) cultural inequities and distrust.

**Discussion:**

The COVID-19 pandemic has contributed to the stigmatization of people living with dementia. Further research is needed to develop, implement, and evaluate interventions targeted towards the different types of dementia-related stigma (including self, public, and structural stigma). Moreover, our findings highlight the need for more collaborative research that prioritizes the lived experience and input of diverse people living with dementia. Research partnerships with diverse people living with dementia are vital to improving future pandemic planning. Only through evidence-informed research and lived experience can we begin to fully address the different types of dementia-related stigma and enhance the quality of life of people living with dementia.

## 1 Introduction

Stigma of dementia is one of the greatest challenges for people living with dementia. Dementia-related stigma can lead to depression, anxiety, low self-esteem, social isolation, poor mental health, and a reduced quality of life for people living with dementia and their care partners (1, 2). Research shows that dementia-related stigma impedes help-seeking, deters healthcare practitioners from providing a dementia diagnosis, hinders access to specialist services (neurologists and geriatricians) (3, 4), and contributes to human rights abuses against people living with dementia (5).

During the pandemic, people with dementia were often depicted in the media as being highly vulnerable, helpless, and at-risk to COVID-19 related mortality (6–8), especially within institutional settings (9, 10). Moreover, pandemic messaging and public health campaigns often failed to recognize that all people were vulnerable to COVID-19. Consequently, studies suggest that the COVID-19 pandemic may have contributed to stigma towards people living with dementia (6).

Prior to the pandemic, existing literature reviews were conducted to understand dementia-related stigma. More specifically, Herrmann and colleagues examined the worldwide literature on dementia-related stigma and identified a critical need for further research to study approaches to reduce stigmatizing perceptions (11). Another review examined interventions to reduce dementia-related stigma and found a variety of education (dispel myths with facts), contact (interact with people with dementia), and mixed (education and contact) interventions ranging from culturally tailored films to intergenerational choirs (12). Recently, Nguyen and Li identified the need for further research to examine public-stigma and self-stigma of dementia within different contexts and cultures (13). Although previous reviews have been conducted, it is difficult to draw any meaningful comparisons or generalizations due to the varying focus of the reviews and different conceptualizations of stigma.

In Goffman’s well-known work, *Stigma: Notes on the Management of Spoiled Identity*, stigma is defined as any attribute that is socially discrediting (14). More recently, Werner and colleagues described stigma of dementia as the emotional, cognitive, and discriminatory attributions held by the public towards people living with dementia (15, 16). In comparison, Corrigan and colleagues define stigma in terms of stereotypes (negative beliefs), prejudice (agreement with beliefs), and discrimination (discriminatory actions or behaviors) through self-stigma (against themselves), public stigma (from a group people including healthcare providers) (17), or structural stigma (institutional policies and practices) that intentionally or unintentionally impact stigmatized individuals (18). Although several definitions of stigma exist, there remains little consensus on its definition. Drawing on Corrigan and colleagues’ framework (self-stigma, public stigma, and structural stigma) (17, 18), this study defines stigma in terms of stereotypes, prejudice, and discrimination against people living with dementia.

Although previous reviews have been conducted on dementia-related stigma, research has only started to address stigma during the COVID-19 pandemic. Substantial work remains to fully understand the COVID-19 effects on dementia-related stigma. Drawing on Corrigan and colleagues’ framework (self, public, and structural stigma), the purpose of this scoping review is to examine the impact of stigma on people living with dementia in the pandemic. As a scoping review, our study adds to the previous knowledge by addressing different types of dementia-related stigma within the context of the pandemic.

## 2 Methods

Our scoping review was guided by Arksey and O’Malley’s scoping review framework (19) and PRISMA guidelines. We registered our scoping review with the Open Science Framework (OSF) on May 29, 2023 (osf.io/5bcgd). We conducted our scoping review by following Arksey and O’Malley’s six steps: i) identifying the research question; ii) examining relevant articles, iii) selecting the articles, iv) extracting the data; v) collating, synthesizing, and reporting the research results; and vi) consulting with stakeholders (19).

### 2.1 Step 1: Identifying the research question

Our study’s main objective was to examine the impact of dementia-related stigma (self-, public, and structural stigma) in the COVID-19 pandemic. To achieve this aim, our study focused on addressing the following question: How did people with dementia experience stigma (self, public, and structural stigma) related to dementia during the COVID-19 pandemic?

### 2.2 Step 2: Examining relevant studies

Relevant studies were identified by searching CINAHL, EMBASE, Medline, PsycINFO, and Web of Science. A search of Google Scholar was also conducted to support supplementary searching to identify any additional relevant literature that may have been missed. More specifically, we conducted this search using our inclusion criteria (such as date restrictions, English language, and peer-reviewed articles) and only reviewed the first 100 articles based on Google Scholar’s relevance sorting function. Studies were also found by scanning the reference lists of the journal articles that were included in our scoping review (n=15) to help ensure that no relevant articles were missed.

Our search timeframe focused on articles published between January 13, 2020 to June 30, 2023. January 13, 2020 was selected as the start date for our timeline because the World Health Organization (WHO) confirmed the first COVID-19 case outside of China on this date.

In conducting systematic literature reviews, researchers often use PICO (Population, Intervention, Comparison, Outcomes) to support their search strategy for relevant information. However, the Joanna Briggs Institute (JBI) recommends the PCC (Population, Concept, Context) tool to help inform the search strategy of scoping reviews. Consequently, we used the PCC tool to aid in our search strategy (documented in **Table 1**).

**Table 1:** Search Strategy.

PCC	Description	Keywords	Databases
Population	People living with dementia	Dementia	CINAHL, EMBASE, Medline, PsycINFO, Web of Science, and Google Scholar.
Concept	Stigma	Stereotypes OR Stigma OR Bias OR Prejudice OR Discrimination	
Context	COVID-19 Pandemic	COVID-19 OR Pandemic OR Coronavirus	

#### 2.2.2 Inclusion and exclusion criteria

Our inclusion criteria consisted of five components: i) full-text, peer reviewed journal articles; ii) articles reporting original research such as qualitative, quantitative, and mixed methods studies; iii) written in the English language; iv) published between January 13, 2020 and June 30, 2023; and v) focus on dementia-related stigma during the COVID-19 pandemic. Our exclusion criteria consisted of the following: i) published in languages other than English; ii) does not report on original research; and iii) does not focus on the study’s aim but addresses other topics such as COVID-19 related mortality.

### 2.3 Step 3: Study selection

**Figure 1:**
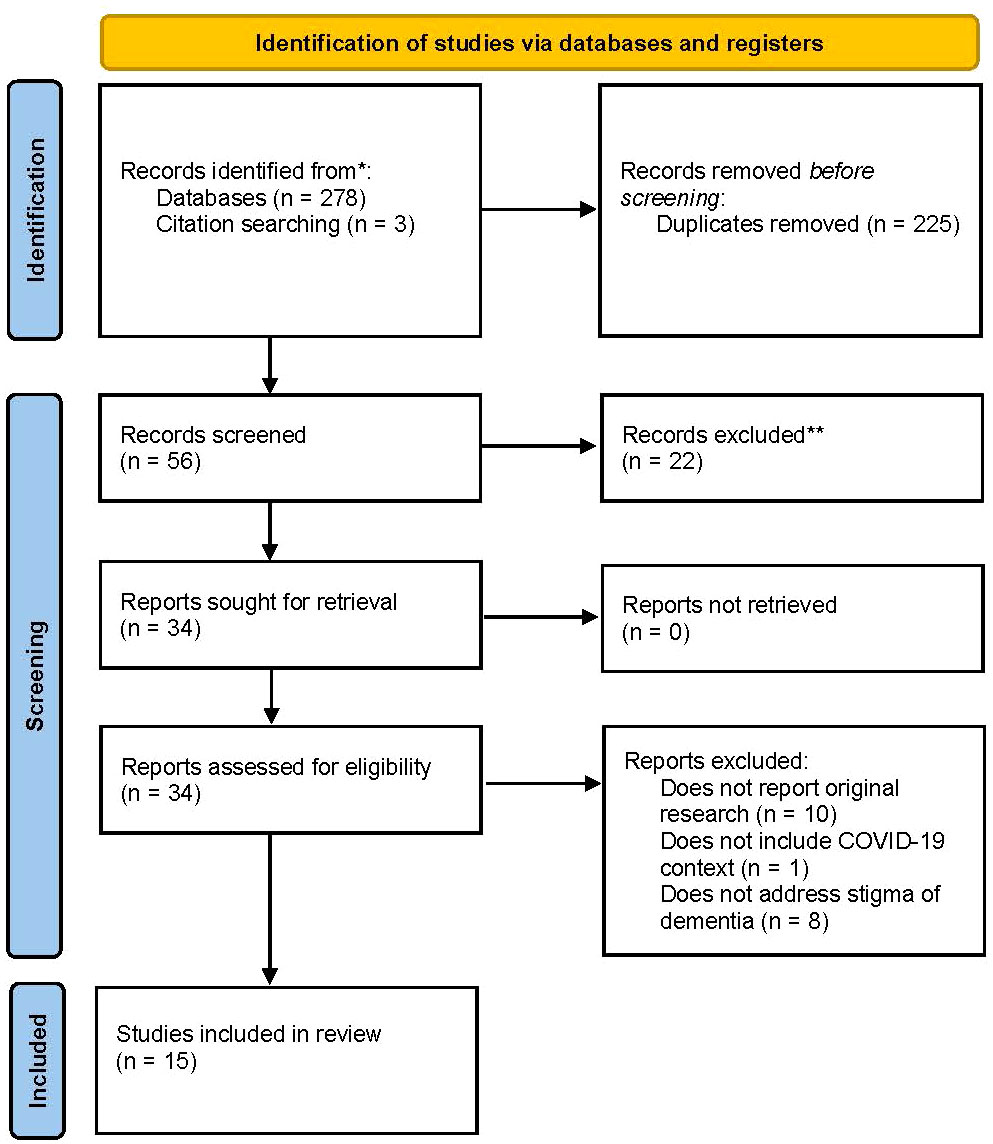
PRISMA flow diagram.

We searched CINAHL, EMBASE, Google Scholar, Medline, PsycINFO, and Web of Science for relevant literature. The search identified 281 articles for potential inclusion in the review. We imported our results (281 articles) into Covidence (https://www.covidence.org/). Covidence was used to help organize and support our collaboration in the study selection and screening process. After 225 duplicates were removed, the titles and abstracts of 56 articles were screened for relevance by two reviewers (JDRB and KN). A total of 22 articles were excluded as they did not meet the inclusion criteria. The full texts of the remaining 34 articles were reviewed, and 19 articles were excluded based on the full-text assessment. The reasons for exclusion were that the articles did not report on original research (n=10), did not focus on stigma of dementia (n=8), or did not address the COVID-19 context (n=1). Any study selection or screening disagreements were resolved through open dialogue between the two researchers, and if required, by consulting the full team to reach a consensus. The final number of documents included in the scoping review was fifteen articles (**Figure 1** shows the PRISMA flow diagram).

### 2.4 Step 4: Extraction of the data

Data from the 15 journal articles were extracted and charted using a data extraction table (**Table 2**). The table included different categories to organize the data such as author/country, purpose, sample, method, stigma type, stigma-related findings, and theme(s). The table was pilot tested by two reviewers (JDRB, KN) to ensure clarity prior to our data extraction process. The two authors conducted the data extraction for all the articles.

**Table 2:** Data Extraction Table.

Authors/Country	Purpose	Sample	Method	Stigma Type	Stigma-Related Findings	Theme(s)
AboJabel (20) Israel	Examines public preferences about whether people with dementia should be prioritized in receiving COVID-19 resources and vaccinations.	309 Israeli Jews aged 40 years and over.	Quantitative research Survey data	Public	Findings: i) dementia related discrimination (public preference favored prioritizing life-saving resources and COVID-19 supports for younger people and older adults without dementia).	Stereotypes and assumptions of dementia.
Armstrong et al. (21) United Kingdom	Explores COVID-19 impact on Black and South-Asian people with dementia.	11 family carers and 4 people with dementia from South Asian or Black communities.	Qualitative research Interviews	Structural	Findings: i) structural distrust (mistrust in government and media), ii) organisational distrust (lack of person‐centred care and inadequate communication with caregivers and care home issues), and iii) cultural and language barriers to accessing supports and services.	Cultural inequities and distrust.
Bacsu et al. (6), Global	Examines stigma of dementia during COVID-19.	1,743 relevant tweets from February 15 to September 7, 2020.	Qualitative research Twitter study	Public	Findings: i) devaluing lives of people with dementia, ii) misinformation about dementia and COVID-19, iii) dementia used for political insults, and iv) challenging stigma of dementia.	Stereotypes and assumptions of dementia.
Bacsu et al. (12) Global	Examines COVID-19 impact on people with dementia.	6,243 relevant tweets from February 15 to September 7, 2020.	Qualitative research Twitter study	Structural	Findings: i) frustration and structural inequities (denied human rights and support services), and ii) despair due to confinement and loss (isolation, cognitive decline, and death).	Human rights and deprived dignity; Disparate access to health and support services.
Bartmess et al. (22), Global	Examines the COVID-19 care concerns of people with dementia.	6,938 tweets from March 17-24, 2020.	Qualitative research Twitter study	Structural	Findings: i) Inadequate and/or no respite care, access to food and medications, internet and technological challenges, information, and isolation, ii) human rights issues (unequal access to care/ventilators, no dignity, pushing do not resuscitate orders).	Human rights and deprived dignity; Disparate access to health and support services.
Cipriani et al. (23), Global	Examines COVID-19 challenges of people with dementia in accessing life-saving supports and services.	Not applicable	Qualitative research Literature review	Structural Public	Findings: i) people with dementia deserve equal right to life and life-saving resources (acute care access and ventilators) during the pandemic; ii) better protocols are required to protect the rights of people with dementia.	Disparate access to health and support services; Stereotypes and assumptions of dementia.
Kimzey et al. (24), United States	Explores impact of nursing students visiting people with dementia online during COVID-19.	10 students, 8 care partners, and 8 persons living with dementia.	Qualitative research Interviews	Structural Public	Findings: i) social contact intervention between students and people with dementia reduced potential for stigma and false assumptions of dementia; and ii) intervention reduced issues of social isolation.	Disparate access to health and support services; Stereotypes and assumptions of dementia.
Lee et al. (25), United States	Examines COVID-19 challenges and supports of Chinese American family care partners of persons with dementia.	26 Chinese American family care partners.	Qualitative research Interviews	Structural	Findings: i) discrimination, denied services, isolation, racism, and distrust due to false assertions (blaming China for spread of COVID-19) made by government officials during pandemic.	Cultural inequities and distrust.
McAiney et al. (26), Canada	Explores COVID-19 impact on lives and connections of people with dementia and their care partners.	10 people living with dementia.	Qualitative research Interviews	Structural Public	Findings: i) fears about physical, cognitive, and social well-being related to contracting COVID-19, ii) frustrations with structural restrictions (concerns about own health and cognition, as well as health of loved ones, and frustration with being unable to visit loved ones or access supports), and iii) perception of people with dementia’s own cognitive decline as a direct result of isolation.	Disparate access to health and support services; Stereotypes and assumptions of dementia.
Nakanishi et al. (27), Japan	Explores neighbourhood social cohesion and dementia-related stigma during COVID-19.	469 Japanese mothers of 16-year-old adolescents.	Quantitative research Survey data	Public	Findings: i) stigma was lower in participants who perceived greater neighbourhood social cohesion, and ii) different cultural perspectives impact perceptions and assumptions of dementia (e.g., dementia relates to negative aging trajectory).	Stereotypes and assumptions of dementia.
Udoh et al. (28), United States	Examines dementia and COVID-19 among older African American adults.	Not applicable	Qualitative research Scoping review	Structural	Findings: i) unequal healthcare access (delays, resources due to lack of health insurance, financial challenges), ii) longer time spent in hospital, and iii) perpetuation of historical and systemic issues in accessing healthcare.	Cultural inequities and distrust.
Werner et al. (29) Israel	Examines public preferences for allocating ventilators to COVID-19 patients with and without Alzheimer’s Disease while differentiating between a young and an old person with the disease	309 Israeli Jewish persons aged 40+ years.	Quantitative Research Survey	Public	Findings: i) dementia related discrimination (71% of participants chose the 80-year-old patient with a diagnosis of Alzheimer’s Disease to be the last to be provided with a ventilator, while remaining quarter were divided between 80-year-old person who was cognitively intact and the 55-year-old person with Alzheimer’s Disease).	Stereotypes and assumptions of dementia.
West et al. (30), United Kingdom	Explores COVID-19 impact on people with dementia and their family carers.	15 people with dementia and their care partners of South Asian and Afro-Caribbean backgrounds.	Qualitative research Interviews	Structural	Findings: i) fear and anxiety, ii) food and eating (shopping access and eating patterns), iii) isolation and identity, iv) community and social relationships, v) adapting to COVID-19, vi) structural barriers from Covid-19 lockdowns (social isolation and support structures), and vii) medical interactions.	Cultural inequities and distrust.
Wilberforce et al. (31), United Kingdom	Explores social care and the engagement of older people with dementia and mental health needs.	22 specialist support workers, 7 managers, 4 homecare staff, 6 service users and carers.	Qualitative research Interviews Focus groups	Public	Findings: i) stereotypes of people with dementia (strange, losing independence, and being a burden); and ii) resistance to support.	Stereotypes and assumptions of dementia.
Yoon et al. (32), Global	Analyzes Twitter data to gain insights to refine interventions for family care partners of people with dementia in COVID-19 pandemic.	58,094 relevant tweets from August 23, 2019, to September 14, 2020.	Quantitative research Twitter study	Public	Findings: i) depression, ii) stigma, iii) loneliness, iv) elder abuse, v) care partner challenges; and vi) stereotypes (COVID-19 victims), vii) care partner coping (resilience, love, and reading books).	Stereotypes and assumptions of dementia.

Compared to systematic reviews, scoping reviews do not evaluate or assess the risk of bias for each study, but rather aim to map and provide an overview of the existing data. Thus, data extraction and charting of the data within scoping reviews does not typically address the methodological quality or risk of bias within each study.

### 2.5 Step 5: Collating, synthesizing, and reporting the results

Drawing on Braun and Clarke’s framework (33), thematic analysis was conducted to examine the patterns and themes related to the impact of stigma of dementia during the COVID-19 pandemic. Specifically, we used inductive thematic analysis by reading through each of the 15 articles. After this initial reading, the relevant stigma-related findings were re-read and annotated line-by-line by 4 coders (JDRB, KN, CS, ZR) to inform the development of our codes. Accordingly, our codes were data driven (inductive) and represented common concepts found in the literature on stigma of dementia during COVID-19. As each article was coded, our code list was updated, and new codes were added to ensure that all data were captured. After our coding was completed, our data were sorted and organized into overarching themes and subthemes that were reviewed by the full team. More specifically, our team reviewed the themes for potential issues with clarity, redundancy, missing themes, and to ensure that the data supported the themes. Consequently, the final theme names were achieved through an iterative process with team discussion to achieve consensus.

### 2.6 Step 6: Consultation with advisory panel

We collaborated with an advisory panel including a person living with dementia and a former family care partner of a person living with dementia. In our advisory panel, the person living with dementia and the former care partner of a family member living with dementia were invited to collaborate to provide their insight and lived experience. For example, the person living with dementia is a nationally recognized advocate and strong speaker committed to educating others on how to reduce dementia-related stigma to improve the quality of life of people living with dementia. She serves on numerous committees and presents regularly on national webinars, conferences, podcasts, and government presentations to advocate and support people living with dementia. Similarly, the former care partner of a family member who lived with dementia is a well-known champion and international advocate of people living with dementia. More specifically, she is a Certified Professional Consultant on Aging (CPCA), a Certified Alzheimer Care Consultant (PAC), as well as a well-known speaker at international webinars, conferences, and podcasts focused on dementia care. She is the president and founder of a consulting firm that provides education and support services a to help people navigate the journey of Alzheimer’s disease or dementia-related illnesses. Our advisory panel provided insight through various stages of the scoping review processes, from identifying the research priorities to supporting the theme development and refinement. More specifically, the advisory panel participated in attending all team meetings, collaborated in identifying the research questions, helped to identify relevant search terms, reviewed the data extraction table, provided input into the theme development, and reviewed the manuscript. In addition, the advisory panel plans to actively engage in the knowledge dissemination activities to share the review’s findings such as co-presenting at conference presentations and webinars. The advisory panel has prior experience collaborating with the lead author in knowledge dissemination activities such as co-presenting at local and national webinars (hosted by the Canadian Consortium on Neurodegeneration in Aging and British Columbia’s Interior Health).

### 2.7 Rigor

Steps were taken to help ensure rigor and trustworthiness in our study. For example, a scoping review protocol was conducted to outline the plans for our review process and reduce the potential for impromptu decision-making that may impact our study’s rigor. Moreover, we comprehensively documented our steps taken in the review process and adhered to a rigorous methodological framework (33) and scoping review guidelines using the Preferred Reporting Items for Systematic Reviews and Meta-Analyses Extension for Scoping Reviews (PRISMA-ScR) checklist.

## 3 Results

### 3.1 Descriptive analysis

A total of 15 articles met our inclusion criteria, including 11 qualitative studies and four quantitative studies (**Table 2**). For example, five studies were based on interviews, one consisted of a combination of interviews and focus groups, two studies were literature reviews, four studies examined Twitter data (three qualitative and one quantitative), and three studies examined survey data. The articles focused on six geographic areas: three from the United Kingdom, three from United States, one from Canada, two from Israel, one from Japan, and five were focused globally.

### 3.2 Self-stigma, public stigma, and structural stigma

From the 15 articles identified, none of the articles discussed self-stigma (internalized), five articles addressed public stigma (external/social), six articles described structural stigma (institutional policies and practices), and four studies described issues related to both public and structural stigma. Public stigma was often described in terms of negative stereotypes, false beliefs, and assumptions towards people living with dementia. In contrast, structural stigma often related to issues of systemic inequities in terms of disparate access to health and support services, human rights issues and denied dignity, and cultural inequities (such as institutional discrimination from governments, media, and healthcare organizations). In comparison, studies that addressed both public and structural stigma addressed both issues of dementia-related stereotypes and assumptions and disparate access to health and support services.

### 3.3 Thematic analysis

**Figure 2:**
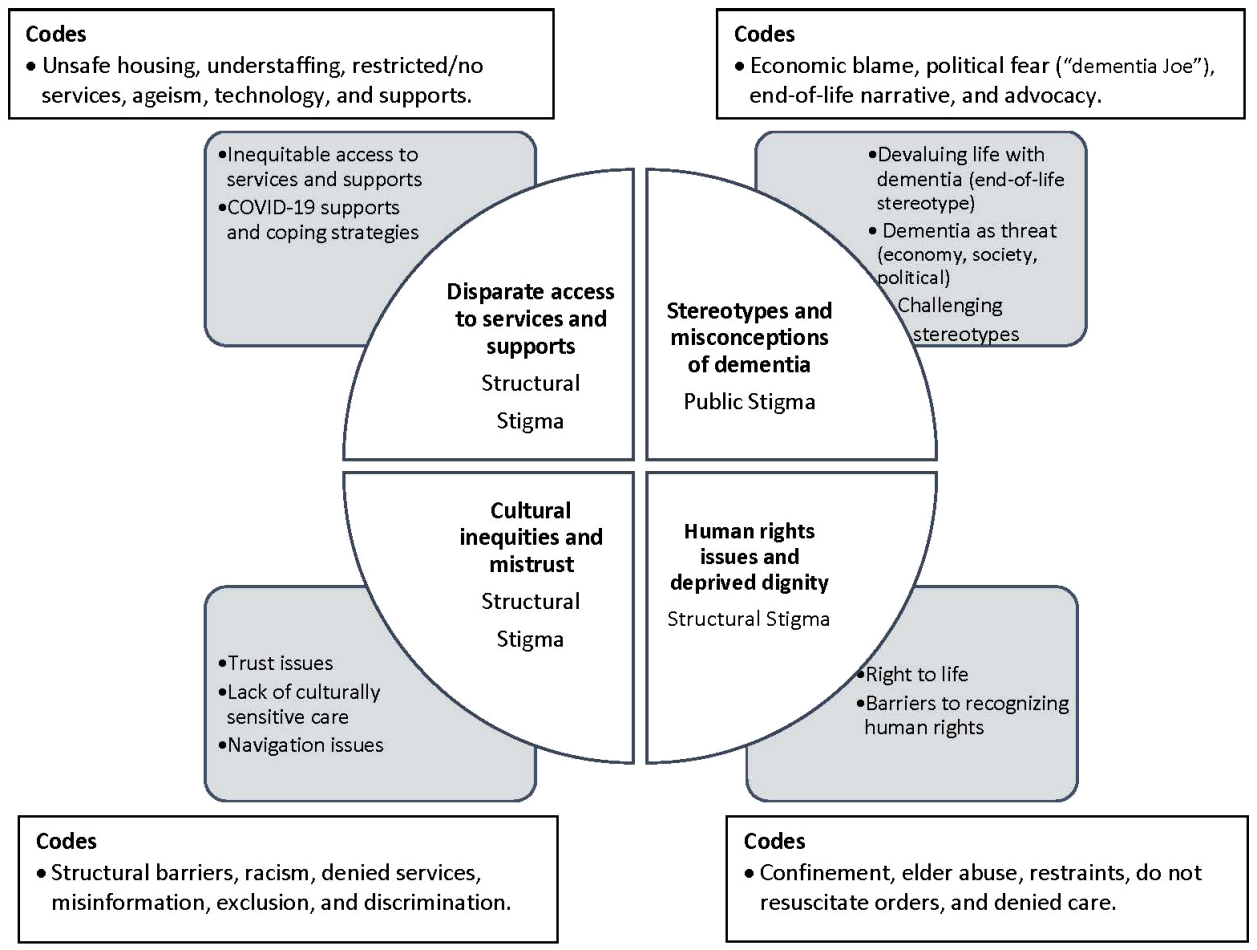
Thematic Map.

Guided by Braun and Clarke’s thematic analysis framework, four primary themes were identified including: 1) COVID-19 stereotypes and assumptions of dementia; 2) human rights issues and deprived dignity; 3) disparate access to health services and supports; and 4) cultural inequities and distrust. A thematic map was created to highlight the themes and show the relationships between the primary themes, subthemes, codes and the types of stigma (**Figure 2**).

#### 3.3.1 COVID-19 stereotypes and assumptions about dementia: public stigma

Public stigma was demonstrated in the theme of COVID-19 stereotypes and assumptions of dementia. Throughout the pandemic, there was a strong stereotype that all people living with dementia were near the end-of-life stage (6, 20). For example, there was a widely held opinion that people with dementia were better off dead and unable to live fulfilling lives in the pandemic (6). Moreover, news media often depicted institutionalized people living with dementia as the highly vulnerable victims of COVID-19 (32). Consequently, research showed that public preference favored not prioritizing life-saving resources and COVID-19 supports (such as ventilators) for older adults living with dementia (20, 29). However, these stereotypes and opinions are problematic because they spread false information, harmful assumptions, and homogenize people living with dementia. Accordingly, McAiney and colleagues note that it is important to recognize that people with dementia are diverse and experience the COVID-19 pandemic differently depending on their unique context and abilities (26).

Dementia-related stereotypes (such as being highly vulnerable and near death) often involved assigning blame and implying that people living with dementia threatened society during the pandemic. For example, people with dementia were held responsible for the poor COVID-19 economy, used to make political ridicule, and blamed for the COVID-19 lockdowns (6). Consequently, people living with dementia were described as facing societal exclusion because of stereotypes such as being strange, losing their independence, and being a burden (31).

Only two studies addressed interventions to challenge stereotypes and false assumptions of dementia. For instance, Kimzey and colleagues reported that a social contact intervention that consisted of online nursing student visits with people living with dementia helped to challenge the students’ stereotypes and assumptions about dementia during the pandemic (24). The second intervention emphasized the importance of using strength-based messaging in providing COVID-19 news media, policies, and public health campaigns. More specifically, this study identified the need for media discourse to focus on precautionary pandemic measures (masking/vaccinations) rather than stereotyping people with dementia as being highly vulnerable to the pandemic (6).

#### 3.3.2 Human rights issues and deprived dignity: structural stigma

Structural stigma (institutional policies and practices) was strongly connected to the theme of human rights and deprived dignity. More specifically, the right to life and dignity were substantial COVID-19 issues impacting people living with dementia (22, 23, 34). Human rights issues were often related to institutional COVID-19 policies, lockdowns, social isolation, confinement, elder abuse, restraint use, overmedication, overcrowding, understaffing, and ageism, especially within institutional settings (34). Cipriani and colleagues assert that the coronavirus brought an increase in social isolation, loneliness, and seclusion for people living with dementia (23). Moreover, inequitable access to ventilators and life-saving services challenged the right to life and raised ethical concerns for people with dementia (22, 23, 34). Family care partners often felt pressured to sign do not resuscitate orders, and people living with dementia were not provided with the same level of care or medical interventions (e.g., ventilators, vaccinations, physician visits, acute care beds, etc.) as other patients (22).

Articles discussed several structural barriers to recognizing the human rights of people living with dementia in the pandemic. These barriers included discriminatory policies (20, 33), systemic ageism, lack of organizational staff and support services (22, 34). Institutional policies such as visitation bans, lockdowns, and exclusion of informal care partners within organizational settings (hospitals, nursing homes, care homes) contributed to inequities impacting people living with dementia. Loss of cognition and death were reported as consequences of forced institutional confinement and COVID-19 lockdowns (34). Consequently, family care partners expressed feelings of despair, anger, and frustration related to structural stigma in terms of theCOVID-19 policies that led to denied dignity and human rights issues impacting people living with dementia (22, 34).

#### 3.3.3 Disparate access to health and support services: structural stigma

Research described structural stigma related to inequitable access to health services and supports for people living with dementia during COVID-19. More specifically, these challenges included disparate access to respite services (22), social support (24, 26), physicians, acute care services, and hospital care (23, 34). Disparate access to care and support was especially problematic during COVID-19 lockdowns for people living with dementia in institutional settings. In particular, COVID-19 policies banned family care partners from accompanying older adults with dementia in hospitals but allowed access to partners of expectant mothers (34). Family care partners also described housing disparities and the lack of safe housing available to people living with dementia because institutions (care homes, nursing homes, etc.) were often inundated with the virus (34).

Although many COVID-19 disparities were identified, studies also shared coping strategies and supports for dealing with COVID-19 disparities. Hope and resiliency were identified as predominant factors for surviving in the pandemic (22, 26). Resiliency was often discussed in terms of one’s ability to adapt to change, manage stress, and have a sense of purpose (22, 26). For example, resiliency was supported by engaging in activities such as meditation, physical activity, cooking, listening to music, playing games, and photography (26).

Adaptation to change and learning new technologies were also identified as supports during the pandemic. Specifically, Kimzey and colleagues found that using new technology (such as Zoom) to engage in online visits enabled people living with dementia to have a sense of social connection to reduce stress and social isolation during COVID-19 (24). Technology also provided access to online support groups, social connection, information hotlines, peer advice, companionship, and enhanced navigation to services during the pandemic (22). However, technology also presented challenges related to inaccessibility and difficulties with user-friendliness for some people living with dementia (23).

#### 3.3.4 Cultural inequities and distrust: structural stigma

Structural stigma was further identified in the theme of cultural inequities and issues of distrust. For example, older African American adults with dementia and COVID-19 were described to experience systemic discrimination, increased delays, and reduced access to healthcare resources such as ventilators, technological supports (e.g., cell phones), transportation, health insurance, and access to intensive care units during COVID-19 (28). Moreover, African American adults often experienced distrust towards the healthcare system and the government due to prejudice, fear, racism, and discrimination (28). Consequently, a lack of trust was related to the information spread by institutions such as the media and the government (21). For example, systemic discrimination and messaging from the government organizations, traditional news media (21, 25), and online social media (27) were reported to heighten the intersection of dementia-related stigma and issues of racism.

Cultural inequities were further perpetuated by the lack of culturally sensitive care (30). Specifically, family care partners of people living with dementia from cultural and ethnic minority groups reported issues of institutional discrimination and exclusion in the healthcare decision-making process in working to support their family members living with dementia. Armstrong and colleagues assert that care partners reported being discriminated against by healthcare providers wrongfully assuming that they could not understand or speak English (21). Moreover, Chinese American care partners described structural discrimination, withholding of healthcare resources, exclusion, and racism which contributed to additional challenges in caring for people living with dementia (25).

Navigation of services and supports was also identified as a barrier for cultural and ethnic minority groups of people living with dementia. More specifically, issues of systemic racism, language barriers, and fear contributed to disparities in navigating dementia care within the healthcare system (21, 25). Accordingly, institutional culturally sensitive policies and anti-racism training are needed to support dementia healthcare and diagnosis (21, 25, 30).

## 4 Discussion

Drawing on Corrigan and colleagues’ (self-stigma, public stigma, and structural stigma) framework (17, 18), we conducted a comprehensive scoping review to examine the impact of stigma towards people living with dementia during the pandemic. Given that our scoping review was restricted to the timeframe of the COVID-19 pandemic, the literature was rather limited. For example, our review identified no studies addressing self-stigma. This finding is consistent with pre-pandemic literature (systematic review of dementia-related stigma) that reported extremely sparse research on self-stigma (13). From the 15 included studies, only 11 qualitative studies and four quantitative studies addressed public stigma, structural stigma, or both types combined. Guided by Braun and Clarke’s thematic analysis framework (33), we identified four primary themes of dementia-related stigma including: COVID-19 stereotypes and assumptions of dementia; human rights issues and deprived dignity; disparate access to health and support services; and cultural inequities and distrust.

Our review found that public stigma (external/social stigma) was strongly connected to the theme of COVID-19 stereotypes and assumptions about dementia. More specifically, our review identified widely held beliefs and stereotypes that people living with dementia were all near the end-of-life stage. This finding is consistent with previous literature which associated people living with dementia with death (near death and living dead) (5). This stereotype is problematic because it spreads false information, homogenizes people with dementia, and can lead to detrimental implications such as inequitable access to health services and lifesaving resources (ventilators, acute care). However, few studies provided interventions to challenge COVID-19 stereotypes against people living with dementia (6, 24). Addressing dementia-related public stigma is critical to improving interactions with healthcare providers, access to services, and receiving timely diagnoses for individuals with dementia. Consequently, further research is required to identify and evaluate interventions to address public stigma and challenge stereotypes of dementia during the pandemic and beyond.

In contrast to public stigma, structural stigma (unfair COVID-19 policies and practices) was often attached to the theme of human rights violations and denied dignity (22, 23, 34). Specifically, COVID-19 policies such as institutional lockdowns and forced isolation often contributed to issues of neglect, overmedication, dehydration, ageism, restraint usage, confinement, elder abuse, and denied access to healthcare services for people living with dementia (34). Consistent with national (35, 36) and international reports (10, 37), our review suggests that structural stigma in the form of institutional confinement and forced segregation policies contributed to social isolation, cognitive impairment, prohibited family care partners from providing essential care, and hastened mortality for people living with dementia. Moreover, a report by AGE Platform Europe identified a number of human rights issues heightened by COVID-19 policies, including forced confinement, inequitable access to healthcare and life-saving services, and issues of abuse towards older adults with disabilities (38). Accordingly, it is essential that any future pandemic policies are evaluated for structural stigma to ensure the human rights and dignity of people living with dementia are protected, especially within institutional settings.

During the pandemic, structural stigma was further demonstrated by restricted or denied access for people living with dementia to essential health and support services (physician visits, ventilators, family care partners, acute care services, etc.) (22, 23, 34). These disparities in access were often connected not only to structural stigma but also institutional ageism. More specifically, COVID-19 hospital policies would often grant institutional access to partners of pregnant women but not allow hospital access to care partners of people living with dementia (34).

Structural stigma was evident in the theme of cultural inequities and distrust among ethnic and racial minority groups of people living with dementia. For example, our review identified barriers related to systemic racism, discrimination, denied healthcare services, and inadequate culturally sensitive care (21, 25, 27). This finding is consistent with pre-pandemic research that indicates that structural stigma in the form of systemic racism and structural discrimination are entrenched in history and has been negatively impacting people with dementia from ethnic and racial minority groups (39, 40). Accordingly, anti-racism training, cultural safety education, culturally tailored interventions, and further research are required to support equitable dementia healthcare and diagnosis for ethnically and racially diverse people living with dementia during the pandemic and beyond (29).

Our study has important implications for policymakers, practitioners, and community leaders working to address dementia related stigma during COVID-19 and future pandemics, crises, and natural disasters. Specifically, research partnerships with diverse people living with dementia are needed to provide critical insight to reduce dementia-related stigma and inequities in future pandemic and crises planning. Moreover, future research needs to examine how culture/ethnicity, sex/gender, geography (rural, remote, urban), and ageism intersect with the different types of dementia-related stigma during the pandemic. Understanding dementia-related stigma among marginalized groups is essential to developing evidence-informed knowledge to enhance future pandemic policies and crises planning. Only through collaborative research and lived experience can policymakers, practitioners, and researchers begin to address the underlying roots of stigma (self, public, and structural stigma) that fueled inequities towards diverse people living with dementia during the COVID-19 pandemic and beyond.

## 5 Study strengths and limitations

To our knowledge, this study is the first review to examine the impact of stigma on people living with dementia during the COVID-19 pandemic. Understanding stigma of dementia is critical because it can lead to detrimental implications that hinder a timely dementia diagnosis and reduce quality of life for people living with dementia. However, our scoping review is not without limitations. For instance, it is important to note that scoping reviews do not involve evaluating or assessing the quality of the research. As a result, our ability to make strong implications about the methodological rigor of the included studies may be limited.

In addition, the focus on peer-reviewed, original research articles may have excluded relevant gray literature and preprint studies. Consequently, future scoping reviews may consider including gray literature and preprint databases. However, there has been a high retraction rate documented amongst preprint articles due to ethical concerns and study misconduct, especially during the COVID-19 pandemic (41).

## 6 Conclusion

The COVID-19 pandemic has contributed to the stigmatization of people living with dementia. Although several articles described COVID-related stigma of dementia, only a few articles identified strategies to reduce public stigma during the pandemic. Further research is needed to develop, implement, and evaluate and interventions targeted towards the different types of stigma (including self, public, and structural). Moreover, our findings highlight the need for collaborative research and partnerships that prioritize the lived experience of diverse people living with dementia. Research partnerships and lived experience are critical to enhance future pandemic planning. Only through evidence-informed research and lived experience can we begin to fully address stigma and improve the quality of life of people living with dementia.

## Author contributions

JB: Conceptualization, Writing – review & editing, Data curation, Formal analysis, Funding acquisition, Investigation, Methodology, Supervision, Writing – original draft. RS: Conceptualization, Writing – review & editing. KN: Writing – review & editing, Formal analysis. ZR: Writing – review & editing, Formal analysis. CS: Writing – review & editing, Formal analysis. CW: Writing – review & editing, Formal analysis. MN: Writing – review & editing, Formal analysis.
